# TLR2, TLR4 AND MyD88 Mediate Allergic Airway Disease (AAD) and *Streptococcus pneumoniae*-Induced Suppression of AAD

**DOI:** 10.1371/journal.pone.0156402

**Published:** 2016-06-16

**Authors:** Alison N. Thorburn, Hsin-Yi Tseng, Chantal Donovan, Nicole G. Hansbro, Andrew G. Jarnicki, Paul S. Foster, Peter G. Gibson, Philip M. Hansbro

**Affiliations:** The Priority Research Centre for Healthy Lungs, Hunter Medical Research Institute and The University of Newcastle, Newcastle, New South Wales, Australia; Queen's University Belfast, UNITED KINGDOM

## Abstract

**Background:**

Exposure to non-pathogenic *Streptococcus pneumoniae* and vaccination are inversely associated with asthma. Studies in animal models demonstrate that airway administration of *S*. *pneumoniae* (live or killed), or its vaccines or components, suppresses the characteristic features of asthma in mouse models of allergic airway disease (AAD). These components could be developed into immunoregulatory therapies. *S*. *pneumoniae* components are recognized by Toll-like receptors (TLR) 2 and TLR4, and both induce inflammatory cell responses through the adaptor protein myeloid differentiation primary response gene 88 (MyD88). The involvement of TLR2, TLR4 and MyD88 in the pathogenesis of AAD and asthma is incompletely understood, and has not been studied in *S*. *pneumoniae*-mediated suppression of AAD. We investigated the role of TLR2, TLR4 and MyD88 in the development of AAD and *S*. *pneumoniae*-mediated suppression of AAD.

**Methods and Findings:**

OVA-induced AAD and killed *S*. *pneumoniae*-mediated suppression of AAD were assessed in wild-type, TLR2^-/-^, TLR4^-/-^, TLR2/4^-/-^ and MyD88^-/-^ BALB/c mice. During OVA-induced AAD, TLR2, TLR4 and MyD88 were variously involved in promoting eosinophil accumulation in bronchoalveolar lavage fluid and blood, and T-helper type (Th)2 cytokine release from mediastinal lymph node T cells and splenocytes. However, all were required for the induction of airways hyperresponsiveness (AHR). In *S*. *pneumoniae*-mediated suppression of AAD, TLR2, TLR4 and MyD88 were variously involved in the suppression of eosinophilic and splenocyte Th2 responses but all were required for the reduction in AHR.

**Conclusions:**

These results highlight important but complex roles for TLR2, TLR4 and MyD88 in promoting the development of OVA-induced AAD, but conversely in the *S*. *pneumoniae*-mediated suppression of AAD, with consistent and major contributions in both the induction and suppression of AHR. Thus, TLR signaling is likely required for both the development of asthma and the suppression of asthma by *S*. *pneumoniae*, and potentially other immunoregulatory therapies.

## Introduction

Asthma is a chronic allergic airways disease (AAD) characterized by airway inflammation and airway hyperresponsiveness (AHR). The incidence of asthma has increased dramatically over the past three decades although disease incidence has now plateaued [1]. The reasons for the increased incidence remain controversial, however, alterations in exposure to microbes during the induction and development of the disease have been widely postulated to be involved [2, 3]. This potentially occurs through altered stimulation of the innate immune system. Pathogen associated molecular patterns (PAMPs) are recognized by pattern recognition receptors (PRRs) such as Toll-like receptors (TLRs). TLRs are expressed on antigen presenting cells, such as macrophages and dendritic cells (DCs). TLR engagement leads to nuclear factor (NF)-κB and/or interferon regulatory factor (IRF)3/7-induced production of inflammatory mediators including TNFα, IL-1β, IL-6, IFNα/β and monocyte chemotactic protein (MCP)-1, which attempt to control infection, as well as anti-inflammatory molecules such as IL-10 [4, 5]. TLR2 and TLR4 are two of the major TLRs involved in the recognition of major bacterial components [6]. TLR engagement likely plays a major role in directing T cells responses and the development of asthma. For example, a polymorphism in TLR2 has been associated with asthma [7–9], and Hammad *et al*., showed that TLR4 expression on lung structural cells, but not DCs, is necessary and sufficient for the induction of AAD [10]. However, much remains to be uncovered of the role of TLRs in asthma pathogenesis. These studies have lead to the investigation of modulating TLRs in asthma. Some have shown that TLR2 and TLR4 agonists may be beneficial in asthma [2, 11, 12], whereas others show that some TLR4 agonists such as lipopolysaccharide (LPS) exacerbate disease [13]. Thus, there is a need to further investigate the contribution of TLR responses in asthma and their potential for therapeutic modulation.

Several recent studies by us, and others, have highlighted the potential use of *S*. *pneumoniae* as an immunoregulatory therapy for asthma [2, 14–19]. We have shown that *S*. *pneumoniae* infection, whole killed bacteria, components, and vaccines suppress the characteristic features of AAD in mice. This includes substantial reductions in eosinophil accumulation in bronchoalveolar lavage fluid (BALF) and blood, Th2 cytokine release from mediastinal lymph nodes (MLNs) and splenocytes and AHR [2, 14–19]. The mechanisms underlying suppression involve the induction of regulatory T cells (Tregs) and the modulation of DCs and natural killer T cells. However, the innate recognition pathways involved in *S*. *pneumoniae-*mediated suppression of AAD that could be manipulated through the development of immunoregulatory components of this bacterium have not been characterized.

The *S*. *pneumoniae* cell wall components lipoteichoic acid, lipopeptides and peptidoglycan are recognized by TLR2 [20–22]. *S*. *pneumoniae* cell wall phosphorylcholine and the exotoxin, pneumolysin are recognized by TLR4 [23, 24], although there is some controversy. It is also known that both TLR2 and TLR4 are involved in controlling *S*. *pneumoniae* infection and that they play a partly overlapping and redundant roles [25]. In addition, the common TLR adaptor protein myeloid differentiation primary response gene 88 (MyD88) is absolutely required for the control of the infection [26].

Since TLR2 and TLR4 are important in innate immunity and asthma, and recognize components of *S*. *pneumoniae*, we hypothesized that these receptors play an important role in the development of AAD and *S*. *pneumoniae*-mediated suppression of AAD. Here, we investigated the involvement of TLR2, TLR4 and MyD88, in ovalbumin (OVA)-induced AAD and *S*. *pneumoniae*-mediated suppression of disease features. We used wild type (Wt) mice and mice deficient (^-/-^) in TLR2, TLR4, TLR2 and 4, or MyD88, and assessed the development of AAD and whole killed *S*. *pneumoniae* (KSpn)-mediated suppression of AAD. We found that TLR2, TLR4 and MyD88 were variously important for the development of inflammation and AHR in OVA-induced AAD. Conversely we also found roles for TLR2, TLR4 and MyD88 in *S*. *pneumoniae*-mediated suppression of inflammation and AHR in AAD.

## Methods

### Animals

Six-eight week-old female BALB/c mice were obtained from the Animal Breeding Facility at The University of Newcastle. TLR2^-/-^, TLR4^-/-^, TLR2/4^-/-^ and MyD88^-/-^ mice on a BALB/c background were provided by the Australian National University (Canberra, Australia). All mice were maintained under specific pathogen free and controlled environmental conditions. Procedures were approved by the Animal Ethics Committee of The University of Newcastle.

### AAD

**Fig 1:**
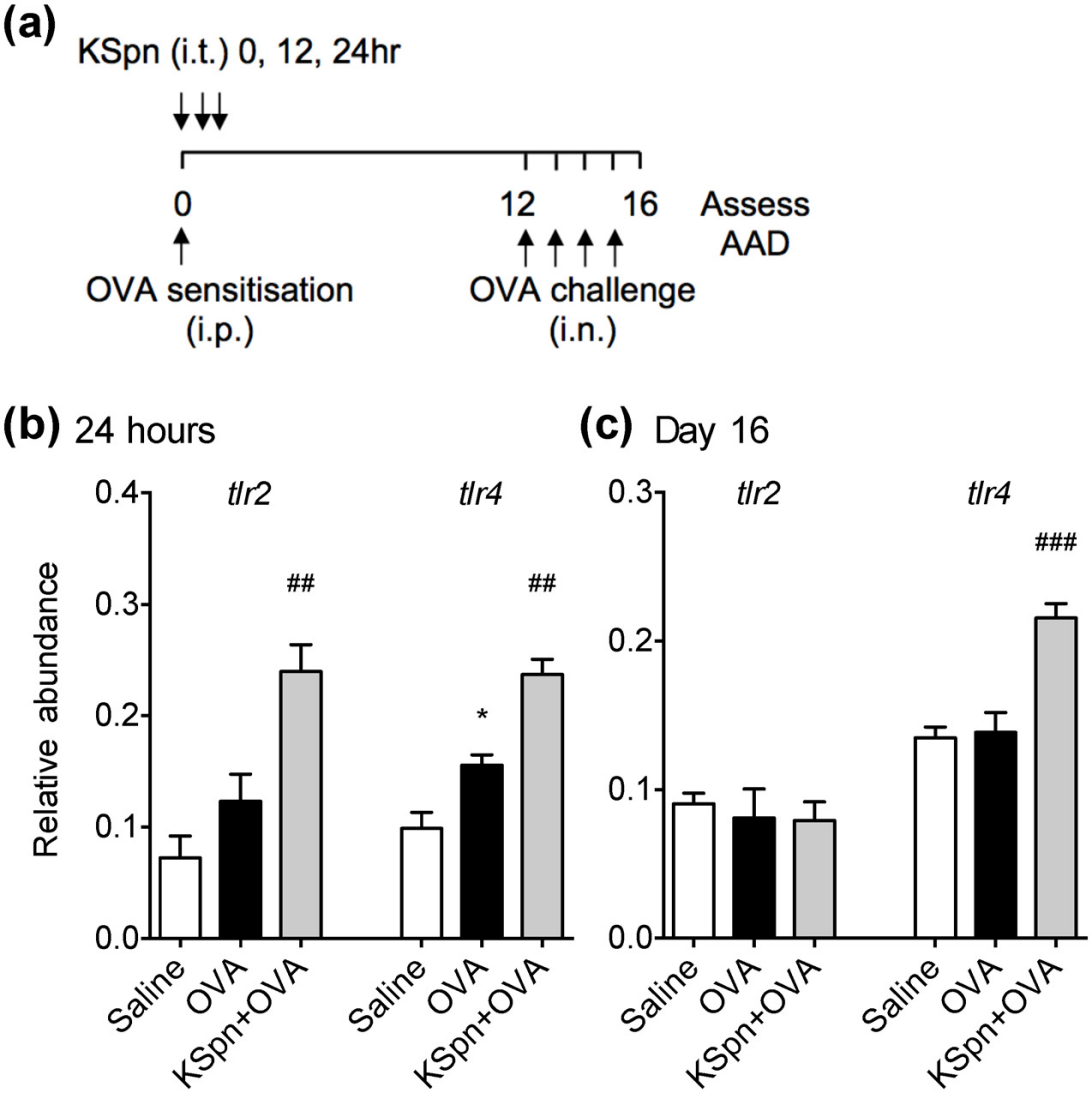
TLR2 and TLR4 mRNA expression in the lung in AAD and KSpn-induced suppression of AAD. Six-week old BALB/c mice were sensitised and challenged with OVA to induce AAD (A). Some groups were administered KSpn i.t. during sensitization. Lungs were collected 24 h after sensitization or on day 16, after the development of AAD. TLR2 and TLR4 gene expression after 24 h (B) or on day 16 (C). Data represent mean ± SEM, n = 6. Significance is represented by * *P* <0.05, (Saline v OVA groups), ##*P* < 0.01, ### *P* < 0.001 (OVA v KSpn+OVA).

The induction of AAD was performed using established techniques as previously described [17, 18, 27–29]. Mice were sensitized to OVA (i.p.; day 0; 50 μg; Sigma-Aldrich, St. Louis, MO) with Rehydragel (1 mg; Reheis, Berkeley Heights, NJ) in sterile saline (200 μl) (Fig 1A). Mice were challenged by intranasal (i.n.) droplet application of OVA (day 12–15; 10 μg in 50 μl sterile saline) under isofluorane anesthesia. Control mice received saline sensitization and OVA challenge. AAD was assessed on day 16.

### Ethanol-killed *S*. *pneumoniae*

During sensitization to OVA, mice were treated with ethanol-killed *S*. *pneumoniae* (2x10^5^ cfu) in sterile saline (30 μl; three doses 12 h apart) by intratracheal (i.t.) administration under intravenous alfaxan anesthesia as previously described (Fig 1A) [16].

### Real-time PCR

For analysis of TLR2 and TLR4 gene expression, total RNA was prepared from whole lungs by TRIzol extraction and cDNA was generated. Real-time RT-PCR was performed as previously described [27, 30], with relative abundance determined by comparison with the reference gene hypoxanthine-guanine phosphoribosyltransferase. The following primer pairs were used: *Tlr2* F: TGTAGGGGCTTCACTTCTCTGCTT, R: AGACTCCTGAGCAGAACAG CGTTT, *Tlr4* F: ATGCATGGATCAGAAACTCAGCAA, R: AAACTTCCTGGG GAAAAACTCTGG.

### Assessment of airway inflammation

BALF was collected as previously described and differential leukocyte counts were determined from a total of 250 cells [31–33].

### Blood collection

Whole blood was collected by cardiac puncture and blood smears prepared as previously described [19].

### T-cell cytokine release

Single cell suspensions were prepared from mediastinal lymph nodes (MLNs) and spleens by pushing through 70 μm sieves and red blood cells lysed. Then 1 x 10^6^ cells/well in 96 well U-bottomed plates were cultured in RPMI media supplemented with 10% FCS, HEPES (20 mM), penicillin/streptomycin (10 μg/ml), L-glutamine (2 mM), 2-mercaptoethanol (50 μM), sodium pyruvate (1 mM). Cells were stimulated with OVA (200 μg/ml) and cultured for 4 (MLNs) or 6 (spleen) days (5% CO_2_, 37°C). Supernatants were collected and stored at -20°C until analysis. Cytokine concentrations in cell culture supernatants were determined by ELISA (BD Pharmingen, San Diego, CA) [19, 27].

### AHR

AHR was assessed as previously described [34–36]. Briefly, anesthetized and tracheotomized mice were cannulated and connected to inline aerosol and ventilator apparatus. Changes in airway function following challenge with increasing doses of aerosolized methacholine (1.25, 2.5, 5 and 10 mg/ml) were assessed by analysis of pressure and flow waveforms, and transpulmonary resistance and dynamic compliance were determined.

### Data analysis

Data were analysed using GraphPad Prism (GraphPad Software, CA) and are represented as the mean ± the standard error of the mean (SEM). One-way ANOVA with Dunnett’s post-test was used to determine significance between data with multiple comparisons. Unpaired Student’s t-test was used to determine differences between two groups. One-way repeated measures ANOVA and Bonferroni’s post-test were used to determine significance for AHR data. *P* < 0.05 was considered statistically significant.

## Results

### Effects of AAD and administration of KSpn on TLR2 and TLR4 mRNA expression in the lung

In this study we used established models of OVA-induced AAD and KSpn-mediated suppression of AAD [16, 19]. We first assessed the expression of *Tlr2* and *Tlr4* mRNA in the lung tissues of Wt mice in these models. Mice were sensitized and challenged with OVA to induce AAD (Fig 1A). TLR mRNA expression during sensitization and after challenge was assessed. There were no changes in *Tlr2* expression in AAD (OVA groups) compared to non-allergic (Saline) controls (Fig 1B and 1C). By contrast, *Tlr4* expression increased 24 h after OVA sensitization but returned to control levels after airway challenges.

In *S*. *pneumoniae*-induced suppression of AAD, mice were treated with KSpn intratracheally then sensitized and challenged with OVA to induce AAD. The expression of *Tlr2* significantly increased 24 h after KSpn treatment and OVA sensitization (KSpn/OVA), but not after challenge, compared to untreated allergic (OVA) controls (Fig 1B and 1C). In addition there were significant increases in *Tlr4* expression following KSpn treatment and OVA sensitization, which was sustained after OVA challenge.

### Roles of TLR2, TLR4 and MyD88 in AAD and KSpn-mediated suppression of eosinophils in BALF in AAD

**Fig 2:**
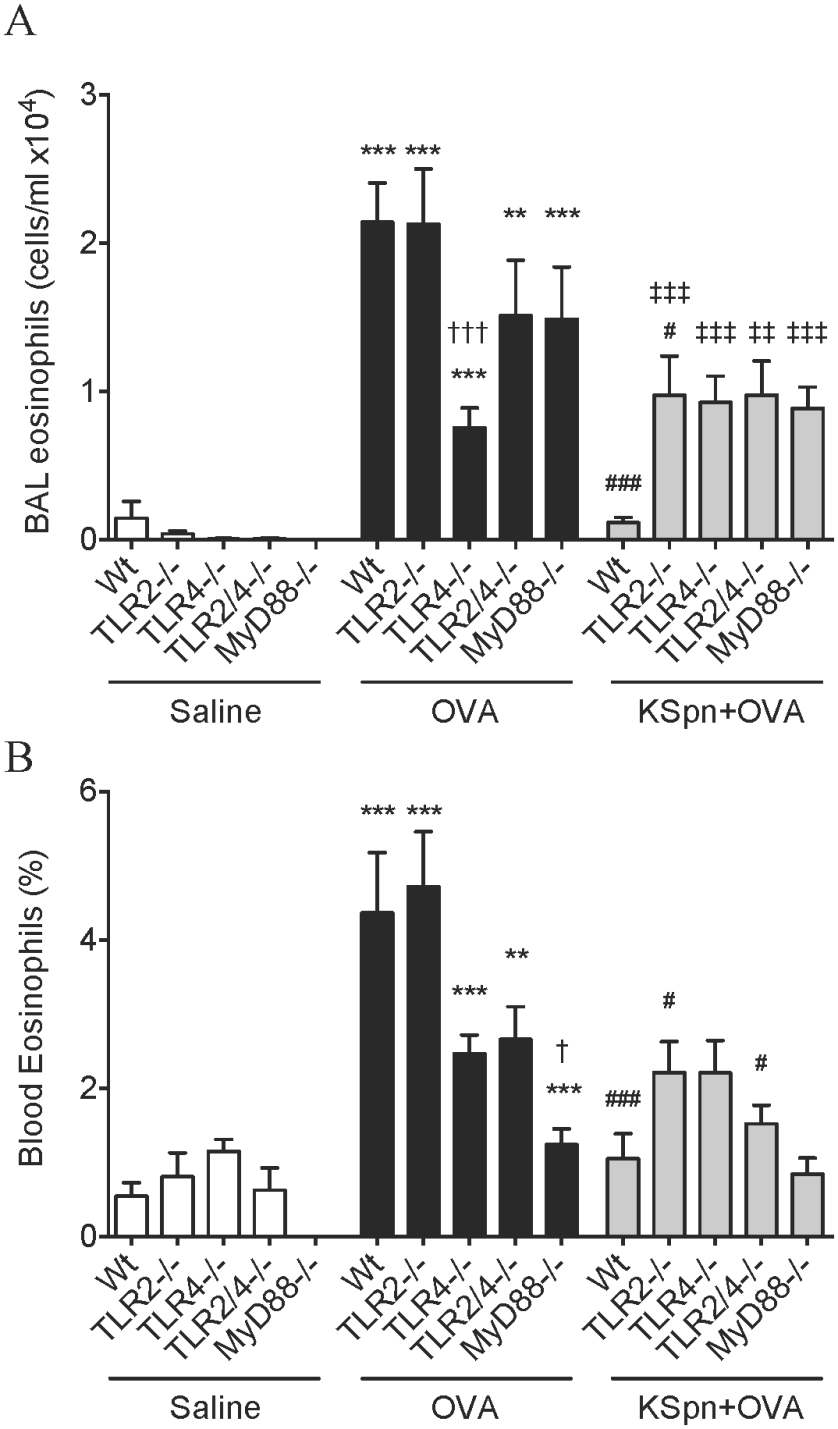
Airway and blood eosinophilia in AAD and KSpn-induced suppression of AAD in MyD88 and TLR deficient mice. Six-week old BALB/c Wt, MyD88^-/-^, TLR2^-/-^, TLR4^-/-^ and TLR2/4^-/-^ mice were sensitized and challenged with OVA to induce AAD. Some groups were administered KSpn i.t. during sensitization. Eosinophil numbers in BALF (A) and percentage in blood (B) were determined. Data represent mean ± SEM, n = 8. Significance is represented by **P < 0.01, ***P < 0.001 (Saline v OVA groups of the same strain), #*P* < 0.05, ###*P* < 0.001 (OVA v KSpn+OVA groups of the same strain), †*P* < 0.05, †††*P* < 0.001 (Wt v -/- between OVA groups) and ‡‡*P* < 0.01, ‡‡‡*P* < 0.001 (Wt v -/- between KSpn+OVA groups).

We then assessed the contribution of TLR2 and TLR4 to AAD and KSpn-mediated suppression of AAD using TLR2^-/-^, TLR4^-/-^ and TLR2/4^-/-^ mice. In addition, we used mice deficient in the TLR2 and TLR4 adapter protein MyD88 (MyD88^-/-^). The induction of AAD was characterized by significant increases in the numbers of eosinophils in the BALF compared to the respective non-allergic controls, in all strains of mice (Fig 2A). Notably, the number of eosinophils in TLR4^-/-^ mice was attenuated compared to Wt mice, indicating that the infiltration of these cells into BALF is partially dependent on TLR4.

As we have shown previously [16], the administration of KSpn led to a substantial and significant reduction in the number of eosinophils in the BALF of Wt mice with AAD compared to untreated Wt allergic controls. KSpn administration also partially but significantly reduced eosinophil infiltration into the airways of TLR2^-/-^ mice compared to untreated TLR2^-/-^ allergic controls. This indicates that TLR2 partially mediates the protective effects of KSpn on BALF eosinophils. However, administration of KSpn did not affect eosinophil infiltration in TLR4^-/-^, TLR2/4^-/-^ or MyD88^-/-^ mice compared to their respective untreated allergic controls. Importantly nevertheless, the affect of KSpn on eosinophil infiltration in Wt mice was significantly greater than in TLR2^-/-^, TLR4^-/-^, TLR2/4^-/-^ and MyD88^-/-^ mice.

### Roles of TLR2, TLR4 and MyD88 in AAD and KSpn-mediated suppression of eosinophils in the blood in AAD

We also assessed the affects of TLR2, TLR4 and MyD88 on eosinophilia in the blood in AAD. AAD resulted in a significant increase in the percentage of eosinophils in the blood compared to the respective non-allergic controls, in all strains of mice (Fig 2B). However, the eosinophil percentage in MyD88^-/-^ mice was attenuated compared to Wt mice. There was also a non-statistically significant trend toward less eosinophils in the blood of TLR4^-/-^ and TLR2/4^-/-^ mice.

As shown previously [16], administration of KSpn led to a significant reduction in eosinophil percentage in the blood of Wt mice compared to untreated Wt controls. Administration of KSpn also significantly reduced blood eosinophils in TLR2^-/-^ and TLR2/4^-/-^ mice compared to the respective untreated allergic controls. However, KSpn had no affect in TLR4^-/-^ or MyD88^-/-^ mice. Notably, assessment of TLR2/4^-/-^ mice showed that TLRs were required for the suppression of eosinophils in BALF due to the absence of TLR4, and in the blood due to the absence of TLR2.

### Roles of TLR2, TLR4 and MyD88 in AAD and KSpn-mediated suppression of IL-5 and IL-13 release from MLN T cells in AAD

**Fig 3:**
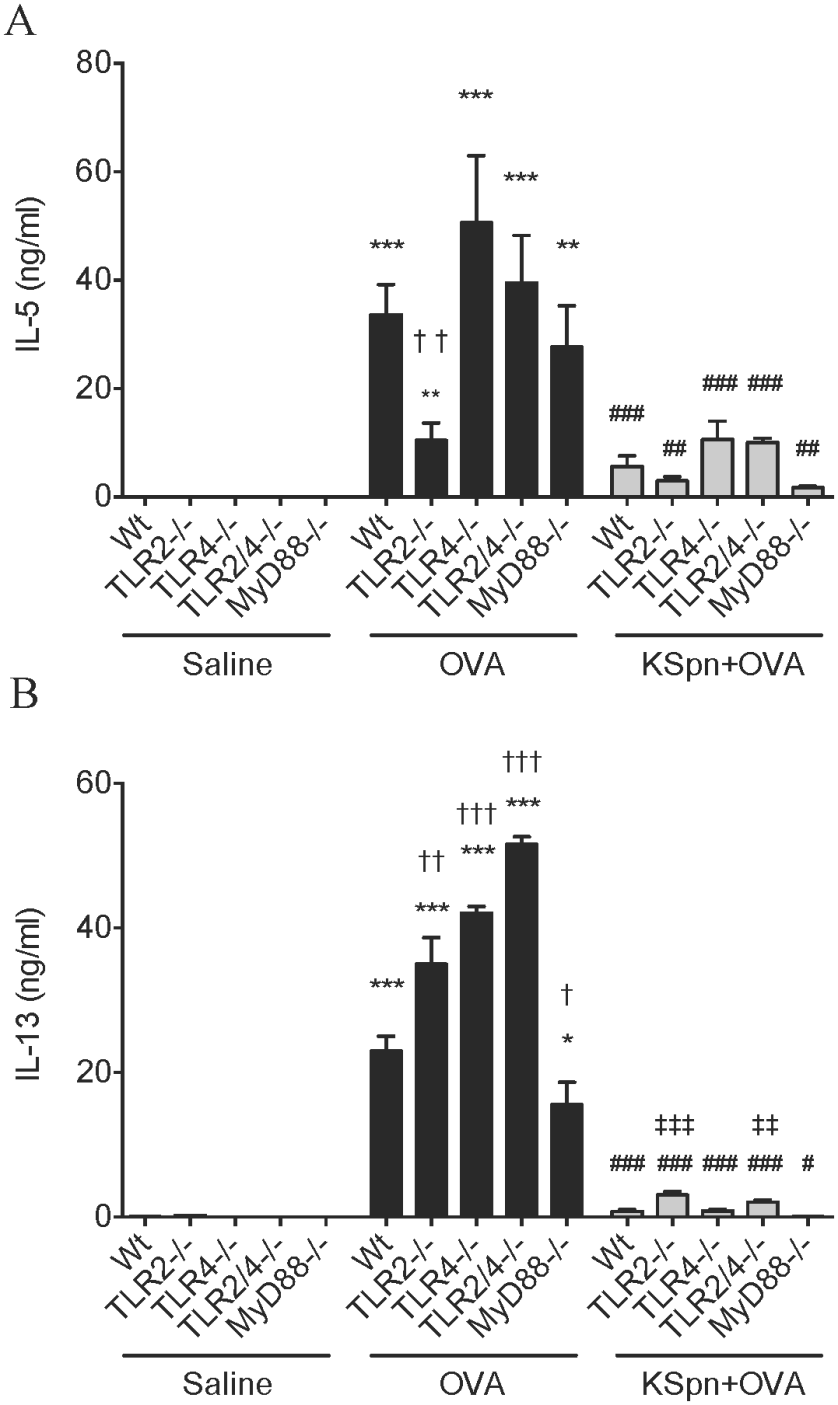
IL-5 and IL-13 release from MLN T cells in AAD and KSpn-induced suppression of AAD in MyD88 and TLR deficient mice. Six-week old BALB/c Wt, MyD88^-/-^, TLR2^-/-^, TLR4^-/-^ and TLR2/4^-/-^ mice were sensitized and challenged with OVA to induce AAD. Some groups were administered KSpn i.t. during sensitization. IL-5 (A) and IL-13 (B) release from MLN T cells was determined by ELISA. Data represent mean ± SEM, n = 8. Significance is represented by *P < 0.05, **P < 0.01, ***P < 0.001 (Saline v OVA groups of the same strain), #*P* < 0.05, ##*P* < 0.01, ###*P* < 0.001 (OVA v KSpn+OVA groups of the same strain), †*P* < 0.05, ††*P* < 0.01, †††*P* < 0.001 (Wt v -/- between OVA groups) and ‡‡*P* < 0.01, ‡‡‡*P* < 0.001 (Wt v -/- between KSpn+OVA groups).

We then assessed the contribution of TLR2, TLR4 and MyD88 on IL-5 and IL-13 release from MLN T cells in AAD and in KSpn-mediated suppression. AAD was characterized by significant increases in IL-5 and IL-13 release from MLN T cells compared to the respective non-allergic controls, in all strains of mice (Fig 3A and 3B). However, IL-5 levels were substantially attenuated in TLR2^-/-^ mice. IL-13 levels were attenuated in MyD88^-/-^ but actually increased in TLR2^-/-^, TLR4^-/-^ and TLR2/4^-/-^ mice compared to allergic Wt controls.

The administration of KSpn substantially suppressed IL-5 and IL-13 release from MLN T cells in all strains compared to their respective untreated allergic controls.

### Roles of TLR2, TLR4 and MyD88 in AAD and KSpn-mediated suppression of systemic IL-5 and IL-13 release from splenocytes in AAD

**Fig 4:**
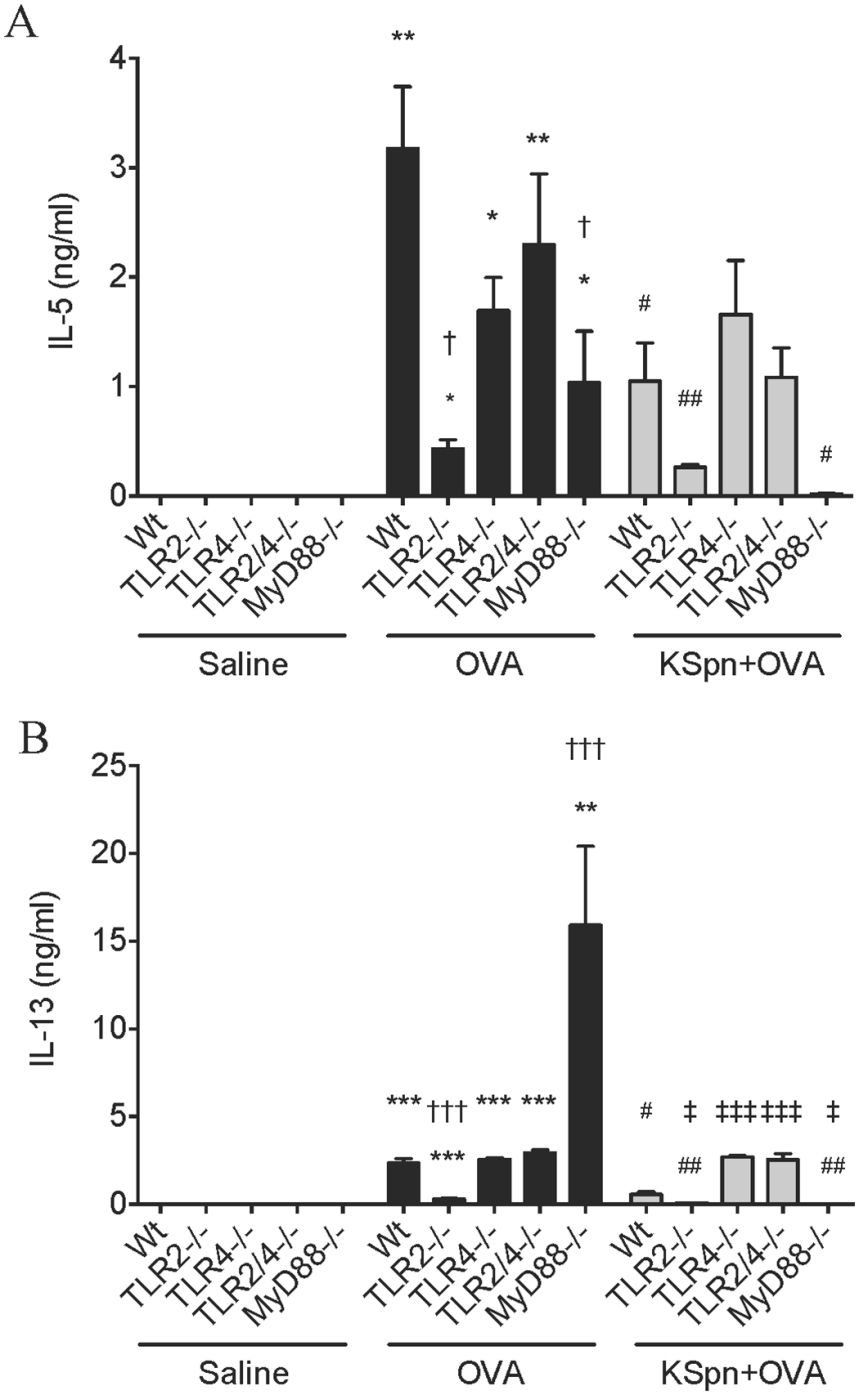
IL-5 and IL-13 release from splenocytes in AAD and KSpn-induced suppression of AAD in MyD88 and TLR deficient mice. Six-week old BALB/c Wt, MyD88^-/-^, TLR2^-/-^, TLR4^-/-^ and TLR2/4^-/-^ mice were sensitized and challenged with OVA to induce AAD. Some groups were administered KSpn i.t. during sensitization. IL-5 (A) and IL-13 (B) release from splenocytes was determined by ELISA. Data represent mean ± SEM, n = 8. Significance is represented by *P < 0.05, **P < 0.01, ***P < 0.001 (Saline v OVA groups of the same strain), #*P* < 0.05, ##*P* < 0.01, ###*P* < 0.001 (OVA v KSpn+OVA groups of the same strain), †*P* < 0.05, ††*P* < 0.01, †††*P* < 0.001 (Wt v -/- between OVA groups) and ‡*P* < 0.05, ‡‡‡*P* < 0.001 (Wt v -/- between KSpn+OVA groups).

We then assessed the contribution of TLR2, TLR4 and MyD88 to systemic IL-5 and IL-13 release from splenocytes in AAD and in KSpn-mediated suppression. AAD was characterized by increases in IL-5 and IL-13 release from splenocytes compared to the respective non-allergic controls in all strains of mice (Fig 4A and 4B). However, IL-5 levels were substantially attenuated in TLR2^-/-^ and MyD88^-/-^ mice compared to Wt allergic controls. IL-13 levels were also attenuated in TLR2^-/-^ mice but in contrast were substantially increased in MyD88^-/-^ mice.

As shown previously [16], administration of KSpn suppressed IL-5 and IL-13 release from splenocytes in allergic Wt mice compared to untreated allergic controls. KSpn also suppressed IL-5 and IL-13 release in TLR2^-/-^ and MyD88^-/-^ mice compared to their respective allergic controls. However, administration of KSpn had no affect on the release of these cytokines in TLR4^-/-^ or TLR2/4^-/-^ mice.

### Roles of TLR2, TLR4 and MyD88 in AAD and KSpn-mediated suppression of AHR in AAD

To assess the contribution of TLR2, TLR4 and MyD88 to physiological outcomes in AAD we investigated their roles in AHR in AAD and in KSpn-mediated suppression.

**Fig 5:**
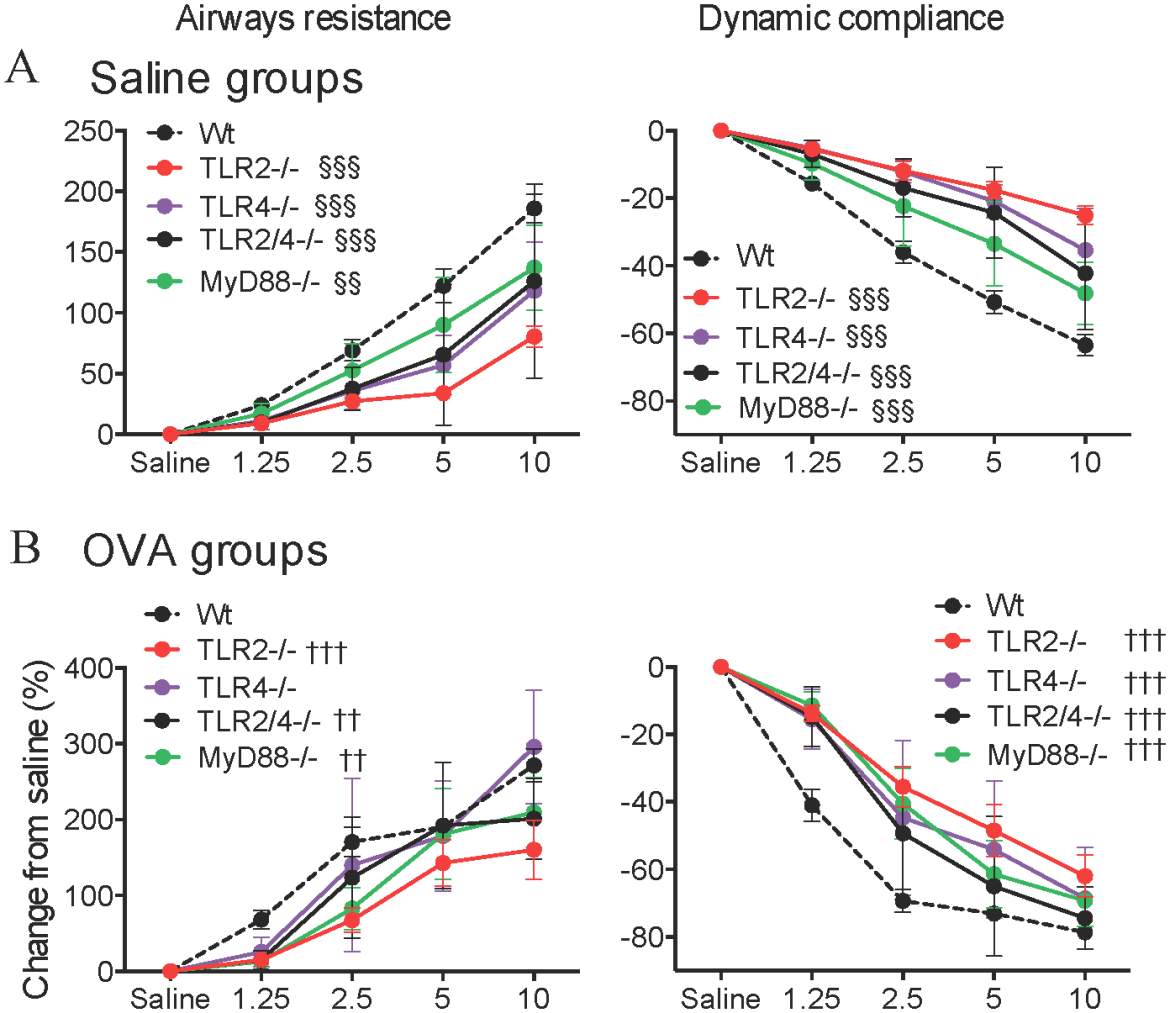
Airway responsiveness at baseline and hyperresponsiveness in AAD in MyD88 and TLR deficient mice. Six-week old BALB/c Wt, MyD88^-/-^, TLR2^-/-^, TLR4^-/-^ and TLR2/4^-/-^ mice were sensitized and challenged with OVA to induce AAD. AHR in terms of airway resistance and dynamic compliance in saline sensitized mice (A). AHR in terms of airway resistance and dynamic compliance in OVA sensitized mice (B). Data represent mean ± SEM, n = 8. Significance is represented by §§*P* < 0.01, §§§*P* < 0.001 (Wt v -/- between Saline groups) and ††*P* < 0.01, †††*P* < 0.001 (Wt v -/- between OVA groups).

AHR was measured in terms of airway resistance and dynamic compliance in response to increasing doses of methacholine. In non-allergic controls, airway responsiveness was attenuated in TLR2^-/-^, TLR4^-/-^, TLR2/4^-/-^ and MyD88^-/-^ compared to Wt mice (Fig 5A). When AAD-induced AHR was established, airway resistance and dynamic compliance were attenuated in all knockout mice compared to allergic Wt controls, with the exception of resistance in TLR4^-/-^ mice (Fig 5B). Nevertheless, the development of AAD did lead to significant increases in airways resistance and decreases in dynamic compliance compared to the respective non-allergic control, in all strains.

**Fig 6:**
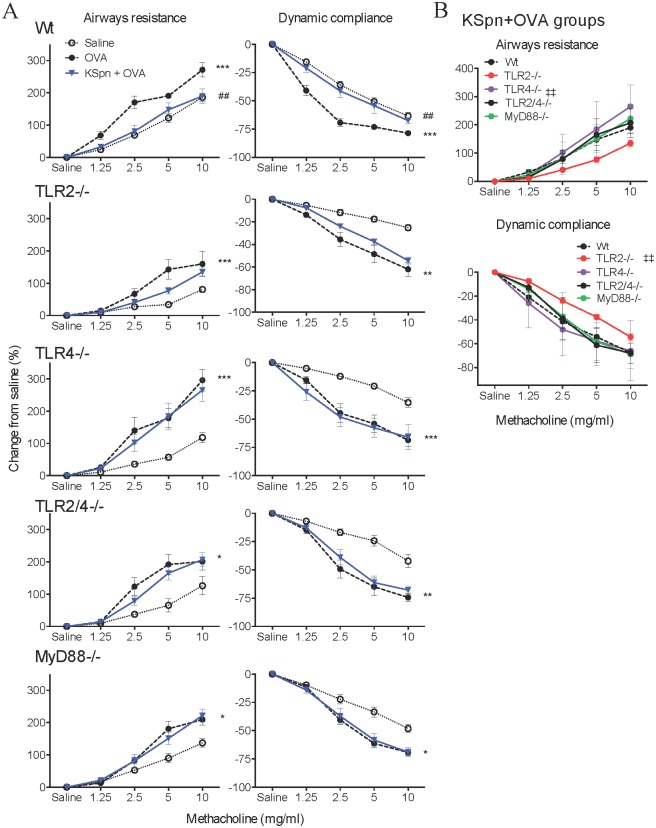
Airway responsiveness at baseline and hyperresponsiveness in KSpn-induced suppression of AAD in MyD88 and TLR deficient mice. Six-week old BALB/c Wt, MyD88^-/-^, TLR2^-/-^, TLR4^-/-^ and TLR2/4^-/-^ mice were sensitized and challenged with OVA to induce AAD. Some groups were administered KSpn i.t. during sensitization. The effect of KSpn on AHR in terms of airway resistance and dynamic compliance in individual mouse strains (A) or combined for comparison (B). Data represent mean ± SEM, n = 8. Significance is represented by *P < 0.05, **P < 0.01, ***P < 0.001 (Saline v OVA groups of the same strain), ##*P* < 0.01 (OVA v KSpn+OVA groups of the same strain), ‡‡*P* < 0.01 (Wt v -/- between KSpn+OVA groups).

Confirming our previous observations [16], administration of KSpn in AAD in Wt mice reduced AHR, significantly lowering airway resistance and increasing dynamic compliance to similar levels as the non-allergic controls (Fig 6A and 6B). In contrast, however, administration of KSpn did not affect the development of AHR in TLR2^-/-^, TLR4^-/-^, TLR2/4^-/-^ or MyD88^-/-^ mice compared to their respective allergic controls.

## Discussion

**Fig 7:**
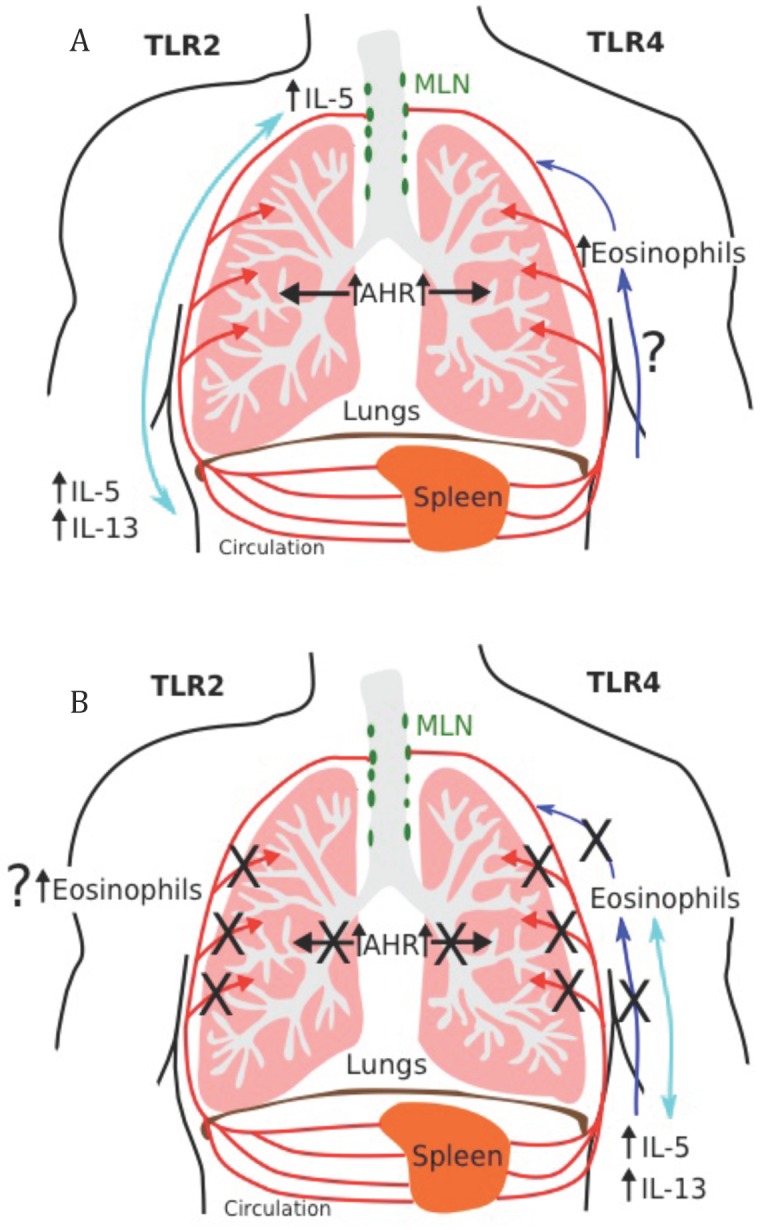
Role of TLRs in AAD and KSpn-mediated suppression. In AAD TLR2 is involved in local IL-5 release from MLN T cells, the systemic release of IL-5 and IL-13 from splenocytes, and the induction of AHR (A). TLR4 is involved in eosinophil influx into the blood (partial) and airways, and the induction of AHR. The presence of either of these TLRs is required to control IL-13 production from MLNs. Many of these effects involve the TLR adaptor protein MyD88, which is required for blood eosinophilia, IL-13 release from MLN T cells and AHR. In KSpn-mediated suppression of AAD TLR2 is involved in the suppression of eosinophil infiltration into the airways and AHR (B). TLR4 is involved in the suppression of eosinophilia in the airways and blood, IL-5 and IL-13 release from splenocytes and AHR. Both TLRs are required for suppression of eosinophils in BALF due to the absence of TLR4, and blood due to the absence of TLR2. MyD88 is required for the suppression of airway and blood eosinophilia and AHR.

Here we demonstrate that TLR2, TLR4 and MyD88 are involved in the development of AAD, and conversely are also required for the suppression of AAD by exposure to bacteria/KSpn. Each of these factors has a differential role in different situations. The induction of AAD lead to increases in eosinophils in BALF and blood, IL-5 and IL-13 production from MLNs and splenocytes, and AHR. Confirming our previous data [16], we again found that these features of AHR were suppressed by the administration of KSpn. We first detected substantial increases in *Tlr2* and *Tlr4* gene expression in the lung during KSpn-mediated suppression of AAD. Then in AAD we identified *inductive* roles for: TLR2 in IL-5 release from MLN T cells, IL-5 and IL-13 release from splenocytes and AHR; TLR4 in eosinophil infiltration into BALF and blood (partial) and AHR, and; MyD88 in blood eosinophilia, IL-13 release from MLN T cells and AHR (Fig 7). In addition, in the absence of both TLR2 and TLR4 there was actually an increase in the release of IL-13 from MLN T cells. This is relevant since local IL-13 release can induce all of the features of AAD/asthma [37–39]. In KSpn-mediated suppression of AAD we identified important *suppressive* roles for: TLR2 in eosinophil infiltration into the airways (partial) and AHR; TLR4 in eosinophilia of the airways and blood, IL-5 and IL-13 release from splenocytes and AHR, and; MyD88 in airway and blood eosinophilia and AHR (Fig 7). Furthermore, data from TLR2/4^-/-^ mice showed that both these TLRs were required for the suppression of eosinophils in BALF due to the absence of TLR4, and blood due to the absence of TLR2. Collectively, these data indicate that KSpn, and its components, can be used in immunoregulatory approaches to suppress AAD, which occurs through the induction of protective TLR responses. This provides further evidence for the use of these and other TLR agonists as asthma therapies [11].

Our analysis of mRNA expression in the lung in AAD showed that only that of *Tlr4* mRNA was increased, and then only during sensitization. However, TLR2 and TLR4 mRNA expression was up-regulated by KSpn, which occurred during sensitization (*Tlr2* and *Tlr4*) and challenge (*Tlr4*). This shows potential roles for TLR4 in allergic sensitization and that both these TLRs are involved in KSpn-mediated suppression of AAD. Furthermore, it indicates the existence of a positive feedback loop where KSpn-induced TLR engagement can lead to increased TLR expression that may be protective in AAD. This is known to occur with the TLR4 ligand lipopolysaccharide, which up-regulates TLR2 in a MyD88-dependent manner [40], and with the TLR2 ligand peptidoglycan that can up-regulate TLR2 expression [41]. TLR2 and TLR4 are expressed by DCs, macrophages, neutrophils, the airway epithelium and some subsets of Tregs, which implicates these cells in many processes that may be manipulated in TLR-directed therapies for AAD/asthma [2, 6, 42, 43].

This study shows that TLR2^-/-^ mice with AAD had reduced IL-5 release from MLNs, IL-5 and IL-13 release from spleen and AHR compared to Wt. In KSpn-mediated suppression TLR2 was implicated in suppressing eosinophils in BALF (partial) and AHR. These outcomes demonstrate that the protective effects of KSpn are only partially dependent on TLR2. *S*. *pneumoniae* infection involves a TLR2 dependent crossing of the epithelial barrier to infect the lung [44]. Therefore in our study the protective effects of KSpn in AAD may not be as pronounced in TLR2^-/-^ mice, because a lack of stimulation of the epithelium that is known to contribute to inflammatory responses in asthma [42]. The altered epithelial barrier in non-allergic TLR2^-/-^ mice may explain why these mice are protected against airway responsiveness compared to non-allergic Wt mice.

TLR4^-/-^ mice are protected against increases in BALF and blood (partial) eosinophils in AAD, highlighting that this receptor is required for eosinophil infiltration in AAD. The decreased levels of eosinophils are not due to reductions in IL-5 and IL-13. TLR4^-/-^ mice have similar levels of airway resistance but increased dynamic compliance compared to Wt mice with AAD. In KSpn-mediated suppression TLR2 was implicated in suppressing blood and BALF eosinophils, splenic cytokine release and AHR. Local cytokine release was unaffected suggesting that the protective effects are systemic through as yet unknown mechanisms, potentially by inducing Tregs and modulating DCs in the periphery [18]. TLR2/4^-/-^ mice with AAD had a trend to decrease eosinophils in BALF and similar decreased levels of eosinophils to TLR4^-/-^ in blood, with evidence of AHR. Administration of KSpn had similar effects to those in TLR4^-/-^ mice.

MyD88^-/-^ mice treated with OVA had decreased eosinophils in BALF (trend) and blood, suggesting additional MyD88 actions that were independent of TLR2/4. MyD88^-/-^ mice with AAD also had a small but significant decrease in IL-13 release from MLN T cells compared to Wt. They also had reduced IL-5 in splenocytes, contrasting with large increases in IL-13 release by splenocytes, and reduced AHR. This provides strong evidence that MyD88 is involved in the control of systemic IL-13 responses. In KSpn-mediated suppression MyD88 was implicated in protection against blood and BALF (partial) eosinophil levels. Our findings are consistent with a similar study that administered LPS/OVA to MyD88^-/-^ mice and showed similar levels of eosinophils to MyD88^-/-^/OVA mice alone [45]. Given that *S*. *pneumoniae* has been shown to activate TLR2, TLR4 and TLR9, the protective effects of KSpn on AAD could be partly driven by a TLR9-MyD88 axis.

Our results with factor deficient mice highlight the differential involvement of TLRs in the development of OVA-induced AAD. Interestingly, the dependence on TLR2 for the induction of IL-5 release from MLN T cells and IL-5 and IL-13 from splenocytes were eliminated with the additional absence of TLR4 (i.e. in TLR2/4^-/-^ mice). The reasons underlying this latter observation are unknown, however, it is likely that redundancy in signaling pathways may be occurring, which is revealed by the absence of both TLRs. Alternate signaling pathways may also be involved. TLR2 and TLR4 can use alternative adaptor proteins such as Toll/interleukin receptor domain-containing adapter-inducing IFN-β (TRIF) or MyD88 adaptor-like (Mal) [46, 47]. We showed further evidence for alternative signaling pathways when the induction of eosinophils in the BALF involved TLR4, but not TLR2 or MyD88. In the absence of MyD88, TLR4 signaling may occur through TRIF or Mal, although there have not as yet been any studies of the links between these other adaptor proteins and IL-5 or IL-13. Our data indicate that other factors may also be involved. In the absence of TLR2, MLN T cell and splenocyte release of IL-5 were reduced but there was no impact on eosinophilia in the BALF or blood. Also, generally in the TLR deficient mice MLN T cell IL-13 levels were increased but splenocyte IL-13 was decreased except for in MyD88^-/-^ mice. This highlights the complexity of TLR responses, and indicates that they have overlapping or unique functions in different situations.

The use of isolated TLR agonists could be used to define their roles in AAD/asthma. Consistent with our findings that KSPn/OVA decreases eosinophils in a TLR2-dependent manner, a single study administered the TLR2/6 agonist, S-[2,3-bispalmitoyiloxy-(2R)-propyl]-Rcysteinyl-amido-monomethoxy polyethylene glycol, conjugated with the antigen peptide (OVA) and challenged in a similar model, which reduced levels of IL-5 in the lung and eosinophils in BALF [48]. Others showed that lipoproteins from pathogenic *S*. *pneumoniae* induces TLR2 to promote the release of TNFα from macrophages during infection [49]. Another study demonstrated that administration of the TLR4 agonist, lipopolysaccharide (LPS), in a mouse model of OVA-induced AAD in Wt mice decreased IL-5 in MLN, IL-13 and eosinophils in the BALF eosinophils [45].

Our study used four strains of TLR/MyD88 deficient mice and compared the effects on AAD and KSpn-mediated suppression of AAD to Wt mice. For some measures the absence of these factors reduced or increased the development of features of AAD, which implicates their involvement in pathogenesis. Nevertheless there were still sufficient alterations in AAD features in factor deficient mice compared to non-allergic controls to enable the assessment of the impact of KSpn. Indeed in some cases KSpn reduced features of AAD in all strains (e.g. Fig 3). Our data in combination with future TLR agonist, human and *in vitro* studies will facilitate the deciphering of the roles of TLRs in *S*. *pneumoniae-*mediated immunoregulation of AAD/asthma.

It is clear from our data that different TLRs have different effects and further investigations are needed to understand this. Clearly individual TLRs are needed for specific processes that are dependent on their known functions and signaling pathways. Collectively our data indicate that different TLRs have different effects in response to different agonists with TLR2 playing more of a role in the induction of AAD and TLR4 more involved in KSpn-mediated suppression. There is also likely to be redundancy, competing or overlapping effects that complicates the understanding of the requirement for each at different stages of the development of disease, i.e. sensitization vs. challenge, and during KSpn-mediated suppression. There is some divorce between the production of pro-AAD cytokines and eosinophil changes and AHR, suggesting that different features are affected at different time points and that different factors are involved. These issues may be addressed by assessing the roles of different factors at different time points and/or using mice in which TLR deficiency is inducible at various stages.

Other TLR or non-TLR pathways may also be involved in KSpn-mediated suppression of AAD. Certain features of AAD were still suppressed by KSpn in the absence of TLR2, TLR4 or MyD88. This again indicates that there may be redundancy in these signaling pathways, other mediators may be involved or that other completely different pathways may be important. For example, KSpn-mediated suppression of eosinophils required TLR4, but not MyD88 and, therefore, TLR4 is signaling through TRIF or Mal in this situation. The suppression of eosinophils in the blood required MyD88, but not TLR2 or TLR4, and may involve recognition by other MyD88-dependent TLRs such as TLR9, which recognizes bacterial DNA [50]. Suppression of IL-5 and IL-13 release from MLN T cells was not TLR or MyD88 dependent, however, suppression of cytokine release from splenocytes required TLR4 and not MyD88 and is likely to occur *via* TRIF.

The independent roles for TLR2 and TLR4 signaling pathways are likely driven by recognition of different KSpn components. Interestingly, TLR2, TLR4 and MyD88 were all required for KSpn-mediated suppression of AHR. This highlights a major involvement of these pathways, which are not redundant, in mediating the suppression of the major physiological precipitation of AAD. These data indicate that in these models AHR is independent of some features of inflammation, which has been shown previously [13]. Collectively, our results show that KSpn-mediated suppression of AAD requires intact TLR2, TLR4 and MyD88 signaling pathways.

TLR2 and TLR4 are expressed by DCs, macrophages, neutrophils, the airway epithelium and some subsets of Tregs, which implicates them in many cellular processes that may be manipulated in TLR-directed therapies for AAD/asthma [2, 6, 42, 43]. Ultimately, TLR signaling can lead to changes in cellular function and pro- or anti-inflammatory responses. For instance, *S*. *pneumoniae*-induced signaling *via* TLR2 and TLR9 enhances phagocytosis and intracellular killing of the bacteria [51, 52]. TLR4 expression on DCs is important in directing Th2 cell responses and inflammation in OVA-induced AAD [43, 53, 54]. Furthermore, some TLR agonists induce anti-inflammatory responses by driving Treg responses [2, 55]. Notably, Tregs are known to be deficient in both number and function in asthmatics and also express TLRs such as TLR4 [2, 56]. Since, Treg are required for KSpn-mediated suppression of AAD and TLR4 is required for attenuation of some features of AAD, Treg expression of TLR4 could play a role in KSpn-mediated suppression of AAD and consequently asthma and this requires further investigation. In addition to circulating cells, the epithelium is now recognized to play a major role in initiating and contributing to Th2-induced responses [42]. Thus, epithelial TLR expression may have important consequences in directing immune responses. Indeed, infection with the bacteria *Klebsiella pneumoniae* up-regulates TLR2 and TLR4 on the airway epithelium [57]. The induction of TLR4 also induces the production of ICOS-expressing CD4 T cells, which can inhibit AAD in a mouse model [58]. Whether TLR4-induced ICOS on CD4 T cells is involved in KSpn-mediated suppression of AAD is unknown. Nevertheless, our studies, and those of others, highlight the important roles for TLR2 and TLR4 on multiple cell types in the orchestration of KSpn-mediated suppression of AAD, which requires further analysis.

In this study we used ethanol killed *S*. *pneumoniae*, which we previously showed suppresses AAD, and contains the TLR ligands, lipoteichoic acid, lipoproteins, peptidoglycan and pneumolysin, which are not destroyed by the alcohol [14]. The use of KSpn does not have the confounding impact of infection and heat killing destroys these TLR agonists. The use of KSpn was the first step in the development of an immunoregulatory therapy and contains all the components of the bacterium, which ensures that all relevant components are present. It is likely that where TLR2 is required for KSpn-mediated suppression, lipoteichoic acid, lipoproteins and peptidoglycan are the signal transducers. Where TLR4 is required, phosphorylcholine and pneumolysin may be the transducers. MyD88 is used by both TLR2 and TLR4 and, therefore, potentially by lipteichoic acid, lipoproteins, peptidoglycan, phosphorylcholine and pneumolysin. Our data indicate that it is these combined TLR engagement events that are important in directing the multi-factorial KSpn-mediated suppression of AAD. We have recently identified two of the components of *S*. *pneumoniae* that are particularly important for suppressing AAD, i.e. the combination of polysaccharide and pneumolysoid (detoxified version of pneumolysin) [17]. In that study pneumolysoid (that signals *via* TLR4), was not effective at reducing features of AAD. However, cell wall components (containing TLR2 ligands) were shown to suppress AAD, suggesting that TLR2 signaling is required for the protective effects. These findings further indicate the importance of TLR2 and TLR4 signaling in mediating the suppressive effects of KSpn on AAD.

In future it will be interesting to extend our studies by investigating the roles of TLRs and impact of KSpn in house dust mite-induced models that involve sensitization direct through the airways. The differential contribution of innate signaling pathways on different cell compartments in AAD and KSpn-mediated suppression could also be investigated using tissue-specific deletion of TLRs or bone marrow chimera experiments as performed by Hammad *et al*., and us [10, 59]. It would also be interesting to assess the role of TLRs in infectious exacerbations of AAD using mouse models [60].

In summary, this study highlights major but complex roles for TLR2, TLR4 and MyD88 in the pathogenesis of AAD and in *S*. *pneumoniae*-mediated suppression of the disease. Each is important in AHR and in the suppression of AHR and there are distinct requirements for TLR2, TLR4 and MyD88 in the development and suppression of inflammation in AAD (Fig 7). We highlight that successful application of KSpn-mediated or other TLR-based immunoregulatory therapies would require patients to have intact TLR signaling pathways for the best outcome. In this regard, polymorphisms in TLR2 have been associated with asthma, implicating the importance of intact TLR signaling pathways [7]. Others have suggested that specific targeting of TLR4 could improve the efficacy of specific allergen immunotherapy [11, 12]. This has been shown with the TLR4 agonist monophosyphoryl lipid (MPL^®^), which has strong immunogenic effects and potential as an adjuvant for allergy vaccines [61]. Since KSpn, targets both TLR2 and TLR4, it may have increased potential for effective suppression of asthma, and *S*. *pneumonia* components or vaccines, may have applicability as human therapies.
